# High mortality among people living with HIV and AIDS accessing care at hospitals in Ghana. Do they report too late?

**DOI:** 10.1186/1471-2334-14-S2-P38

**Published:** 2014-05-23

**Authors:** Thomas Agyarko-Poku, Shella Bawa, Angela El-Aldas, Helena Afriyie Siaw, Yaw Adu Sarkodie

**Affiliations:** 1 Suntreso Government Hospital, Ghana Health Services, Kumasi, Ghana; 2 Ghana AIDS Commission, Accra, Ghana; 3 Department of Clinical Microbiology, School of Medical Sciences, Kwame Nkrumah University of Science and Technology, Kumasi, Ghana

## Introduction

The reported high level of mortality among patients both on clinical care and on Highly Active Antiretroviral Therapy (HAART) in Ghana in recent years has been blamed on an advance presentation of AIDS cases due to delay by patients accessing care at Hospitals. The study hypothesized that HIV and AIDS patients report at the ART clinics with very low CD4 lymphocyte count and determined the CD4 lymphocytes count of HIV positive patient at first presentation in the Ashanti Region of Ghana.

## Materials and methods

This retrospective study reviewed clinical records of 2,971 patients accessing care at 19 ART Clinics in the Ashanti region of Ghana for the period January 2010 to December 2012. The date of first reporting and the level of first CD4 counts were recorded. Socio demographic information was also recorded. Data was analyzed using SPSS version 16.

## Results

More than half (54.9%, 1631/2971) of all HIV patients reviewed presented with CD4 count of less than 250 cells/mm3. Of this number, 37.7% (615/1631) reported with CD4 count less than 50 cell/mm3, 17.3 %(282/1631) with CD4 count of less than 100 cells/mm3, 45.2% (737/1631) with CD4 count of less than 250 cells/mm3. Almost a quarter (23.9%, 710/2971) presented with CD4 count between 250 and 350cells/mm3 with 21.2% (630/2971) reporting with CD4 count of 350 cells/mm3 and above of which only 11.1% ( 70/630) came with CD4 greater than 500cell/mm3. In all more than three quarter (78.8%, 2341/2971) of the patients reported with CD4 count of less than 350cell/mm3.

## Conclusions

The study shows that CD4 lymphocytes count of HIV patients accessing care at Hospitals is very low. Delay reporting might account for this development. Public education on the need to access care at the earliest possible time once tested HIV positive must be intensified. Further study is needed to determine the causes for late presenting at the HIV clinic and address them, since this may account for high mortality among HIV positive patients.

